# Silibinin Potentiates Antimicrobial Action and Reduces Staphyloxanthin in *Staphylococcus aureus*

**DOI:** 10.3390/ph19040643

**Published:** 2026-04-18

**Authors:** José Lima Pereira-Filho, Amanda Graziela Gonçalves Mendes, Carmem Duarte Lima Campos, Viviane da Silva Sousa Almeida, Aleania Polassa Almeida Pereira, Israel Viegas Moreira, Cinara Regina Aragão Vieira Monteiro, Louriane Nunes Gomes, Cristianne Roberta Rhoden, Antonio José Cantanhede-Filho, Lucilene Amorim Silva, Alberto Jorge Oliveira Lopes, Rafael Cardoso Carvalho, Valério Monteiro-Neto

**Affiliations:** 1 Graduate Program in Health Sciences, Federal University of Maranhão—UFMA, São Luís 65080-805, MA, Brazil; jlp.filho@discente.ufma.br (J.L.P.-F.); amanda.graziela@discente.ufma.br (A.G.G.M.); carmem.campos@discente.ufma.br (C.D.L.C.); viviane.almeida@discente.ufma.br (V.d.S.S.A.); polassa.aleania@discente.ufma.br (A.P.A.P.); israel.moreira@ufma.br (I.V.M.); carvalho.rafael@ufma.br (R.C.C.); 2 School of Physical Therapy, Florence University Center, São Luís 65020-490, MA, Brazil; cinara.monteiro@florence.edu.br; 3 Laboratory of Pathology and Immunoparasitology, Center of Biological and Health Sciences, Federal University of Maranhão, São Luís 65080-805, MA, Brazil; louriane.nunes@discente.ufma.br (L.N.G.); lucilene.silva@ufma.br (L.A.S.); 4 Cedro Laboratory, São Luís 65020-570, MA, Brazil; crisrhoden@hotmail.com; 5 Graduate Program in Chemistry, Federal Institute of Scientific and Tecnological Education of Maranhão, São Luís 65030-005, MA, Brazil; prof.antoniofilho@ifma.edu.br

**Keywords:** silibinin, *Staphylococcus aureus*, ciprofloxacin, synergy, antivirulence, staphyloxanthin, biofilm inhibition

## Abstract

**Background/Objectives:** The emergence of methicillin-resistant *Staphylococcus aureus* (MRSA) necessitates innovative strategies to overcome conventional resistance mechanisms. This study investigated the potential of the natural flavonolignan silibinin (SIL) as an antivirulence agent against *S. aureus*, with a particular emphasis on its putative multi-target antibacterial activity and its capacity to potentiate the effects of ciprofloxacin (CIP). **Methods:** The antibacterial and antivirulence properties of SIL were assessed using both in vitro and in silico approaches. The minimum inhibitory concentration (MIC) and minimum bactericidal concentration (MBC) were determined, and its synergistic interaction with CIP was evaluated using checkerboard assays. Inhibition of biofilm formation, as well as disruption of established biofilms, was assessed using an MTT-based viability assay. Staphyloxanthin (STX) inhibition was examined through pigment extraction and spectrophotometric quantification of pathway intermediates. Molecular docking studies were conducted to predict the binding affinities of the compounds to key bacterial targets, while safety was evaluated through haemolytic and cytotoxicity assays. **Results:** SIL exhibited weak to moderate direct antibacterial activity (MICs of 256–512 µg/mL), which is characteristic of many natural product scaffolds. Notably, SIL potentiated the activity of CIP, reducing its MIC by up to fourfold against selected resistant strains of *S. aureus*. SIL significantly inhibited biofilm formation and disrupted established mature biofilms in a strain-dependent manner. In vitro metabolic profiling and in silico analyses provided mechanistic insights into the effects of SIL on STX biosynthesis. Precursor accumulation data suggest inhibition at the diapophytoene desaturase (CrtN) catalytic step, representing a potential mechanism not previously reported for flavonolignans. Molecular docking studies further predicted favourable binding affinities for CrtM and other key targets. Importantly, SIL exhibited no haemolytic activity and low cytotoxicity in macrophages at synergistic concentrations. **Conclusions:** This study provides evidence that SIL functions as a dual-action agent, potentiating ciprofloxacin efficacy while reducing STX production and inhibiting biofilm formation, thereby impairing key virulence mechanisms of *S. aureus*. These findings, together with its favourable safety profile, provide a strong rationale for the development of silibinin-based topical adjuvants to combat drug-resistant *Staphylococcus* infections in humans.

## 1. Introduction

The increasing spread of multidrug-resistant bacteria poses a severe threat to public health and safety. Antimicrobial resistance continues to escalate globally, undermining the effectiveness of life-saving treatments and placing populations at heightened risk. This situation is further exacerbated by the emergence of drug-resistant strains alongside the limited development of new antibiotics [1]. Among priority pathogens, methicillin-resistant *Staphylococcus aureus* (MRSA) is a leading cause of both nosocomial and community-acquired infections and is recognised as one of the most critical resistant pathogens in terms of public health [2,3].

Antivirulence therapy has emerged as a conceptually distinct approach to conventional antimicrobial chemotherapy. By targeting bacterial virulence factors rather than essential growth processes, antivirulence strategies aim to disarm pathogens and render them more susceptible to host immune clearance, while theoretically exerting reduced selective pressure for the development of resistance [4,5,6]. However, as Dickey et al. (2017) emphasised, the distinction between ‘pure’ antivirulence agents and compounds with modest direct antimicrobial activity is often blurred in natural products, which frequently exhibit multiple mechanisms of action [5]. This nuanced perspective is particularly relevant when evaluating plant-derived compounds, such as flavonoids and flavonolignans [5].

The clinical utility of ciprofloxacin (CIP) against *S. aureus* has been markedly diminished by widespread resistance. This is particularly critical in the case of MRSA, in which fluoroquinolone resistance rates frequently exceed 80% in many hospital settings, rendering this class of drugs ineffective for empirical therapy [7]. A key mechanism underlying this resistance is the overexpression of efflux pumps such as NorA, which reduce intracellular antibiotic accumulation [8]. Furthermore, the historical use of CIP has also selected for resistant community-associated MRSA (CA-MRSA) strains, thereby compromising the treatment of common skin and soft tissue infections [9]. Therefore, strategies aimed at inhibiting NorA and reversing this resistance are urgently needed to restore the efficacy of CIP and expand therapeutic options against this pathogen [10].

Beyond efflux-mediated antibiotic resistance, *S. aureus* relies on a broad arsenal of virulence factors to establish and maintain infection. In recent years, antivirulence therapy has attracted increasing interest as an alternative approach aimed at enhancing the susceptibility of pathogens to the host immune system and antimicrobial agents, without exerting the selective pressure that drives resistance development [11]. Among the key virulence determinants, staphyloxanthin (STX), a golden carotenoid pigment unique to *S. aureus*, protects the bacterium against reactive oxygen species (ROS) generated by neutrophils [12].

**Figure 1:**
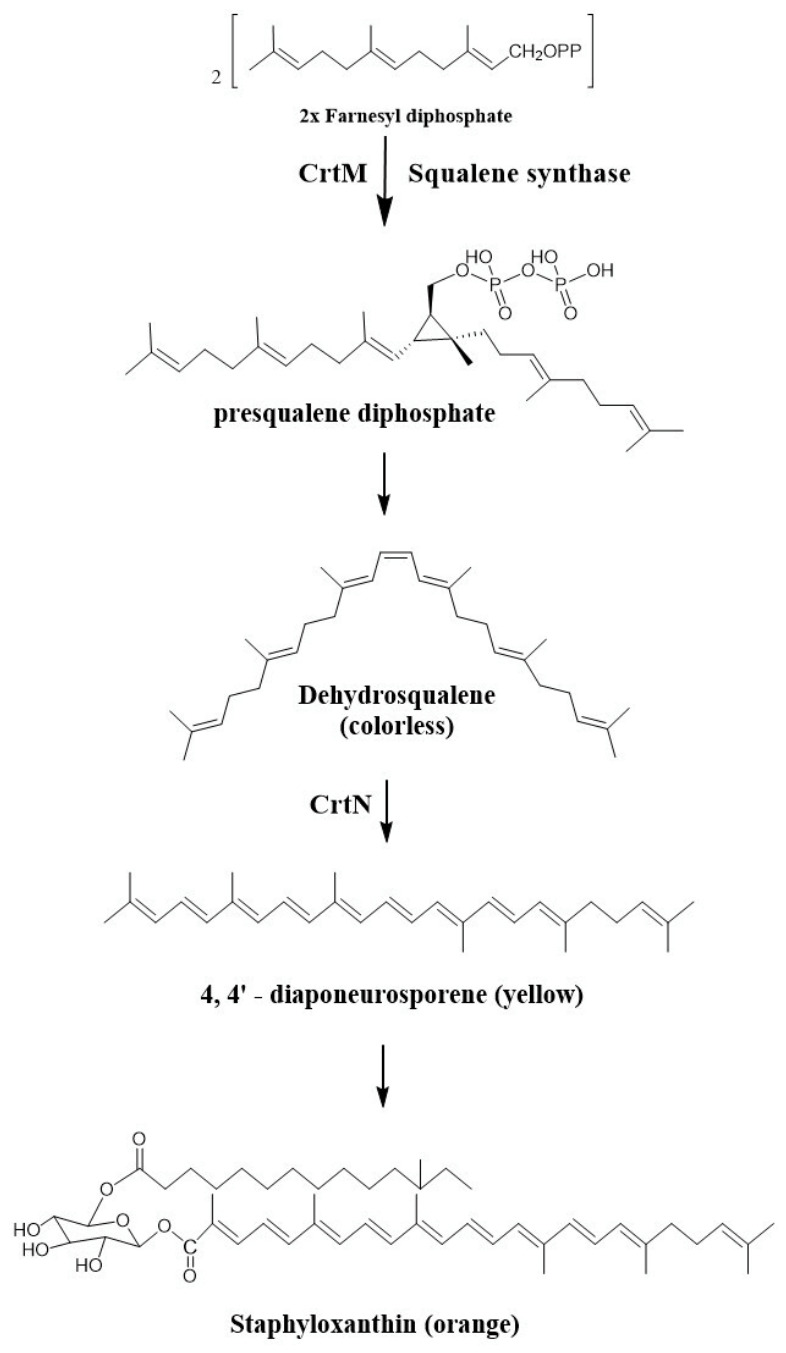
Staphyloxanthin biosynthesis pathway in *S. aureus*. The chemical structures were rendered by the authors using ChemDraw 18.1 (PerkinElmer Informatics, Waltham, MA, USA), adapted from previously published chemical structures [13].

The biosynthetic pathway of STX in *S. aureus* is governed by five key enzymes: dehydrosqualene synthase (CrtM), diapophytoene desaturase (CrtN), diaponeurosporene oxidase (CrtP), glycosyltransferase (CrtQ), and acyltransferase (CrtO) (Figure 1). Disruption of this pathway therefore represents a rational antivirulence strategy, as it may impair bacterial survival within the host and render *S. aureus* more susceptible to immune clearance [13].

Therefore, inhibition of STX biosynthesis has been recognised as a promising strategy for the treatment of MRSA infections, and several inhibitors, including natural products such as eugenol [11], xanthohumol [14], and various flavones [15], have been identified in recent years. However, the identification of novel chemical scaffolds with STX-inhibitory activity remains valuable, particularly those that also target additional virulence mechanisms, such as biofilm formation [13,16].

Natural products, particularly phenolic compounds derived from plants, have garnered renewed interest owing to their structural diversity and broad spectrum of bioactivities [17,18]. Among these, silibinin (SIL), the major flavonolignan from *Silybum marianum* (L.) Gaertn., has attracted attention not only for its well-documented hepatoprotective effects but also for its emerging antimicrobial potential [19].

Unlike many common flavonoids, SIL possesses a unique fused flavonolignan structure that may confer distinct target specificity [19]. Recent evidence suggests that SIL can inhibit bacterial efflux pumps, such as NorA in *S. aureus*, thereby potentially reversing resistance to certain antibiotics [20,21].

While previous studies have reported the antibacterial effects of SIL against clinically relevant bacteria, including *S. aureus* [22,23], these investigations have relied primarily on basic in vitro assays without detailed mechanistic characterisation. Notably, although SIL has demonstrated synergism with β-lactams and aminoglycosides [23,24], its interaction with fluoroquinolones, such as CIP, against Gram-positive pathogens remains unexplored. This represents a significant gap, given the clinical importance of CIP and the potential of SIL to counteract NorA-mediated resistance in *S. aureus*. Furthermore, although limited computational studies have focused on efflux pumps, systematic analyses of SIL binding affinity towards other critical targets (e.g., DNA gyrase, dihydrofolate reductase, and STX biosynthesis enzymes) are still lacking. While STX inhibition has been documented for other classes of natural products, no study has systematically investigated the potential of the flavonolignan SIL to inhibit STX production in *S. aureus*. Moreover, the specific molecular targets within the STX biosynthetic pathway have not been elucidated for any flavonolignan to date.

Therefore, this study aimed to systematically investigate the antibacterial and antivirulence properties of SIL in *S. aureus.* Specifically, we focused on: (i) its direct antibacterial activity and synergism with CIP; (ii) its effects on both early-stage and mature biofilms; (iii) its potential to inhibit STX biosynthesis; and (iv) its binding affinity for key bacterial targets using integrated in silico and in vitro approaches.

## 2. Results

### 2.1. Determination of Minimum Inhibitory and Bactericidal Concentrations and Synergistic Profiling

The direct antibacterial activity of SIL was evaluated against a panel of reference strains and clinical isolates by determining the minimum inhibitory concentration (MIC) and minimum bactericidal concentration (MBC) (Table 1). As shown in Table 1, SIL exhibited weak to moderate antibacterial activity against both reference strains and clinical isolates, with MIC values ranging from 256 to 512 µg/mL. Based on the MBC/MIC ratios, SIL demonstrated bactericidal activity against *S. aureus* ATCC 25923 and MRSA-2 (MBC/MIC = 2), and bacteriostatic activity against MRSA-3.

For context, the MIC and MBC values for the standard antibiotics ciprofloxacin (CIP) and oxacillin (OXA) are provided in Tables S1 and S2.

The interaction between SIL and CIP was evaluated against *S. aureus* strains using a checkerboard assay. The combination exhibited a synergistic effect against *S. aureus* ATCC 25923 (FICI = 0.5) and the clinical isolate MRSA-4 (FICI = 0.375), with the MIC of CIP reduced by up to fourfold in both strains. The SIL/CIP combination showed an additive effect against *S. aureus* ATCC 29213 (FICI = 0.531). When combined with oxacillin (OXA), the interactions ranged from additive to indifferent, depending on the strain (Table 2).

Based on these interaction profiles, specific strains were selected for subsequent antivirulence assays. *S. aureus* ATCC 25923 was chosen as the reference strain, for which the SIL/CIP combination demonstrated clear synergism (FICI = 0.5). Among the clinical isolates, MRSA-4 was prioritised because it exhibited the strongest synergistic interaction with CIP (FICI = 0.375) and a highly resistant phenotype (CIP MIC = 256 µg/mL). This targeted selection enabled the evaluation of the antivirulence potential of SIL and its combination under both standard laboratory conditions and in a clinically relevant, highly resistant isolate.

Given the promising synergistic activity observed, particularly with CIP, we further investigated the potential molecular basis of these effects by performing molecular docking studies against key *S. aureus* targets.

### 2.2. Molecular Docking Predicts Favourable Binding Affinities of Silibinin for Key S. aureus Targets

Molecular docking studies were performed to evaluate the binding affinity of SIL towards key *S. aureus* targets: DNA gyrase B (GyrB), undecaprenyl pyrophosphate synthase (UPPs), dehydrosqualene synthase (CrtM), thymidylate kinase (TMPK), and dihydrofolate reductase (DHFR). The calculated binding free energies (ΔGbind) for SIL and the control compounds are summarised in Table 3.

SIL exhibited favourable binding energies across all targets, with predicted affinities comparable to those of reference compounds for GyrB, UPPs, and CrtM, and lower than the respective controls for TMPK and DHFR (Table 3). For GyrB, SIL demonstrated a ΔG_bind of −8.703 kcal/mol, comparable to that of novobiocin (−8.465 kcal/mol). Similarly, for UPPs, SIL exhibited a binding energy (−8.701 kcal/mol) comparable to that of farnesyl diphosphate (−8.283 kcal/mol) and viridicatumtoxin (−8.231 kcal/mol). A favourable affinity was also predicted for CrtM (ΔG_bind = −10.539 kcal/mol), comparable to those of the known inhibitors B70 (−10.581 kcal/mol) and zaragozic acid A (−10.742 kcal/mol). For TMPK, SIL showed a lower binding affinity (ΔG_bind = −8.542 kcal/mol) than the control T05 (−9.973 kcal/mol). The strongest affinity was observed for DHFR (ΔG_bind = −11.840 kcal/mol), which, although lower than that of trimethoprim (−14.840 kcal/mol), suggests a potential interaction warranting further investigation.

**Figure 2:**
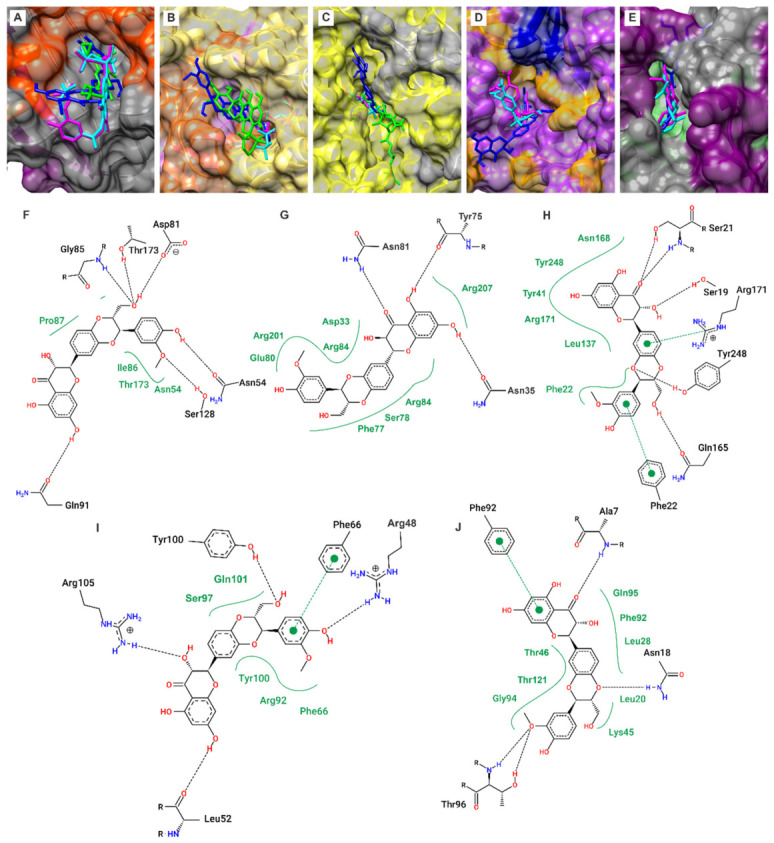
Molecular docking poses and ligand interaction diagrams of SIL (blue) with *S. aureus* targets: (**A**) GyrB; (**B**) UPP; (**C**) CrtM; (**D**) TMPK; and (**E**) DHFR. The positive controls are displayed in green: (**A**) CIP; (**B**) viridicatumtoxin, and (**C**) zaragozic acid A. In magenta, the redocking of the original crystal ligand is shown, including (**A**) novobiocin; (**B**) farnesyl diphosphate; (**C**) B70; (**D**) T05; and (**E**) trimethoprim. The original positions of the native ligands in the crystallographic structures are represented in cyan. Additionally, it presents two-dimensional diagrams of the contacts between SIL and the amino acid residues in the active sites of each target: (**F**) GyrB; (**G**) UPP; (**H**) CrtM; (**I**) TMPK; and (**J**) DHFR. The types of interactions are denoted as follows: dashed black lines indicate hydrogen bonds, solid green lines represent van der Waals interactions, dashed green lines between aromatic rings signify pi–pi interactions, and dashed green lines between an aromatic ring and a cation represent pi-cation interactions.

The reliability of the docking protocol was confirmed by a maximum root-mean-square deviation (RMSD) of 1.16 Å between the crystallographic and redocked native ligands. Analysis of the binding poses revealed that SIL engaged active-site residues across all targets through multiple interaction types (Figure 2). Specifically, SIL formed hydrogen bonds with Asn54, Asp81, Gly85, Gln91, Ser128, and Thr173 in GyrB; Asn35, Asn81, and Tyr75 in UPPs; Ser19, Ser21, Gln165, and Tyr248 in CrtM; Arg48, Leu52, Arg105, and Tyr100 in TMPK; and Ala7, Asn18, and Thr96 in DHFR. These interactions were complemented by multiple van der Waals, π–π, and π–cation interactions with key residues (Figure 2F–J). This network of interactions is consistent with the hypothesis that SIL may act on multiple bacterial targets, a premise that warrants direct enzymatic validation in future studies.

### 2.3. Silibinin Exhibits No Haemolytic Activity

**Figure 3:**
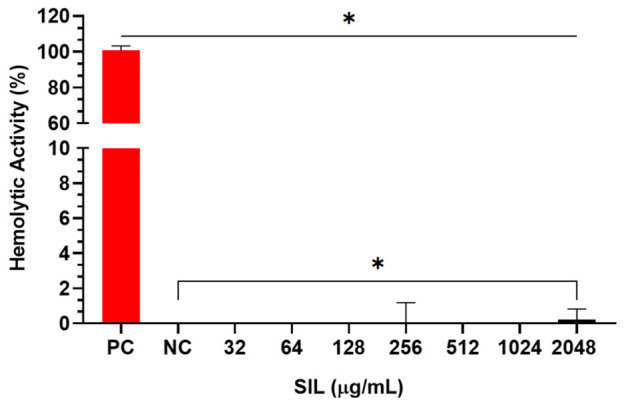
Evaluation of haemolytic activity of SIL in sheep erythrocytes. The percentage of haemolytic activity was compared to that of Triton X-100, which was used as a haemolysis control. Significant differences were determined by one-way analysis of variance (ANOVA) followed by Tukey’s multiple comparison test. Asterisk (*) indicate a *p*-value < 0.05.

Given the biological activities observed, the safety profile of SIL was further assessed by evaluating its potential haemolytic effects on sheep erythrocytes. Haemolysis was quantified by comparing the absorbance values of samples treated with various concentrations of SIL to those treated with 0.1% Triton X-100, used as the positive control. No significant haemolytic activity was observed at any of the tested concentrations of SIL. Even at the highest concentration tested (2048 µg/mL), the haemolysis rate was only 0.20%, indicating that SIL does not compromise erythrocyte membrane integrity (Figure 3).

### 2.4. Cytotoxicity Profiling of Silibinin and Its Synergistic Combination with Ciprofloxacin

**Figure 4:**
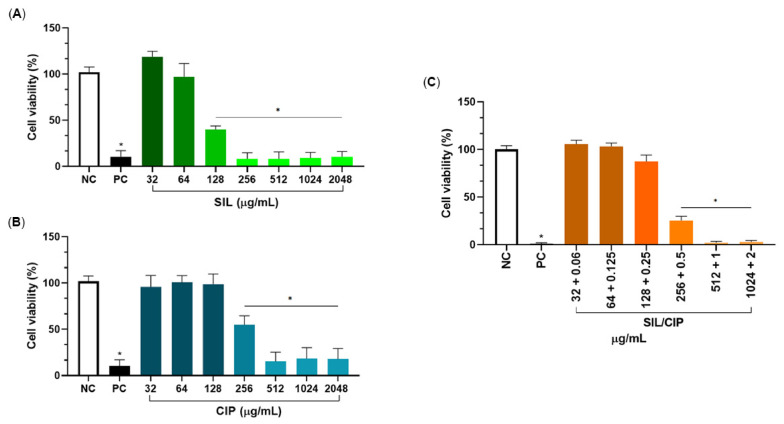
Cytotoxicity profile of (**A**) SIL, (**B**) CIP, and (**C**) their combination in murine macrophages after 48 h of treatment. Significant differences were determined by one-way analysis of variance (ANOVA) followed by Tukey’s multiple comparison test. Asterisk (*) indicate a *p*-value < 0.05.

The cytotoxicity of SIL, CIP, and their synergistic combination was evaluated in RAW 264.7 murine macrophages using an MTT assay. SIL alone exhibited concentration-dependent cytotoxicity, with significant effects observed at concentrations ≥ 128 µg/mL (Figure 4A). Similarly, CIP exhibited cytotoxic effects at concentrations ≥ 256 µg/mL (Figure 4B).

Based on the previously demonstrated synergistic interaction between SIL and CIP against *S. aureus* ATCC 25923, the cytotoxicity of their combination was assessed at synergistic ratios. Notably, the combination treatment containing 128 µg/mL SIL and 0.25 µg/mL CIP, corresponding to their synergistic concentrations, showed no significant reduction in macrophage viability (Figure 4C). These findings indicate that therapeutically effective synergistic combinations maintain a favourable cytotoxicity profile in mammalian cells.

Following the cytotoxicity assays, SIL was further evaluated for its antivirulence activity against both early-stage and mature biofilms.

### 2.5. Silibinin Inhibits Biofilm Formation and Disrupts Mature Biofilms

The antibiofilm potential of SIL was assessed against both early-stage (nascent) and pre-formed mature biofilms of *S. aureus* ATCC 25923 and the clinical isolate MRSA-4, selected based on their synergistic profile with CIP.

**Figure 5:**
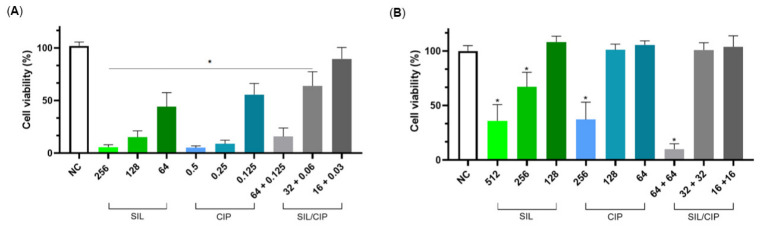
Inhibition of early-stage (young) biofilm formation in *S. aureus* by SIL, CIP, and drug combinations (SIL/CIP). Strains: (**A**) *S. aureus* ATCC 25923 and (**B**) MRSA-4. Significant differences were determined by one-way analysis of variance (ANOVA) followed by Tukey’s multiple comparison test. Asterisk (*) indicate a *p*-value < 0.05.

Against early-stage biofilms, SIL alone significantly reduced biofilm viability at MIC and sub-inhibitory concentrations, although with strain-dependent patterns (Figure 5). For *S. aureus* ATCC 25923, all treatments except the SIL (16 µg/mL) + CIP (0.03 µg/mL) combination at 1/4× MIC produced a statistically significant reduction (*p* < 0.05) in biofilm formation (Figure 5A). The SIL/CIP combination at sub-inhibitory concentrations also effectively inhibited nascent biofilms of this strain. In contrast, the same combination showed no significant activity against nascent MRSA-4 biofilms (Figure 5B).

**Figure 6:**
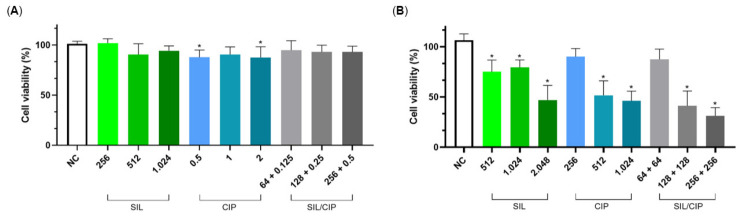
Disruption of pre-formed mature biofilms in *S. aureus* by SIL, CIP, and drug combinations (SIL/CIP). Strain: (**A**) *S. aureus* ATCC 25923 and (**B**) MRSA-4. Significant differences were determined by one-way analysis of variance (ANOVA) followed by Tukey’s multiple comparison test. Asterisk (*) indicate a *p*-value < 0.05.

Against pre-formed mature biofilms, treatment efficacy revealed pronounced strain-dependent effects (Figure 6). For *S. aureus* ATCC 25923, only CIP at concentrations of 256 µg/mL (MIC) and 1024 µg/mL (4× MIC) significantly reduced mature biofilm viability (Figure 6A). In contrast, MRSA-4 mature biofilms exhibited broader susceptibility. SIL monotherapy significantly disrupted mature MRSA-4 biofilms at all tested concentrations (512 to 2048 µg/mL) in vitro. Furthermore, the SIL/CIP combination demonstrated significant activity against MRSA-4 mature biofilms at 128 + 128 µg/mL (2× MIC) and 256 + 256 µg/mL (4× MIC). Importantly, the only treatments that failed to significantly inhibit mature MRSA-4 biofilms were CIP at MIC alone and the SIL/CIP combination at MIC (Figure 6B).

Taken together, the antibiofilm and synergistic activities of SIL, predominantly observed at sub-inhibitory concentrations, support its role as an antivirulence agent beyond direct bactericidal action. To further characterise this potential, we investigated the effect of SIL on staphyloxanthin biosynthesis, a key virulence determinant of *S. aureus*.

### 2.6. Silibinin Inhibits Staphyloxanthin Biosynthesis, Suggesting Interference at the CrtN Catalytic Step

**Figure 7:**
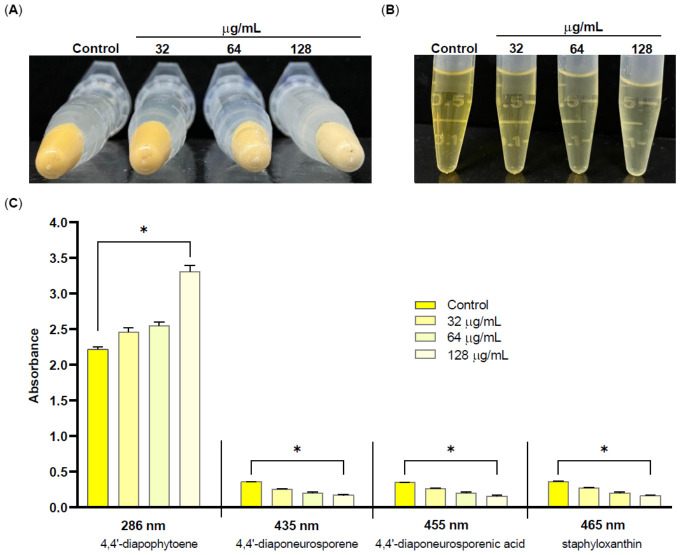
Silibinin inhibits staphyloxanthin biosynthesis and alters carotenoid intermediate profiles in MRSA. Qualitative evaluation of STX production in the presence of increasing concentrations of SIL (32, 64, and 128 µg/mL) in (**A**) bacterial pellets and (**B**) methanol extracts. (**C**) Spectrophotometric quantification of STX intermediates, namely 4,4′-diapophytoene, 4,4′-diaponeurosporene, 4,4′-diaponeurosporenic acid, and STX, extracted from control and SIL-treated MRSA cells (32, 64, and 128 µg/mL). Significant differences were determined by one-way analysis of variance (ANOVA) followed by Tukey’s multiple comparison test. Asterisk (*) indicate a *p*-value < 0.05.

The effect of SIL on STX production was evaluated using phenotypic and metabolic analyses. Qualitative assessment of bacterial pellets from SIL-treated cultures (32, 64, and 128 µg/mL) revealed a concentration-dependent loss of the characteristic golden pigmentation compared with the untreated control (Figure 7A). Methanol extraction of pigments confirmed a dose-dependent decrease in STX production (Figure 7B). At the highest concentration (128 µg/mL), the bacterial pellets appeared light yellow, indicating a substantial impairment of the STX biosynthetic pathway.

To delineate the underlying molecular mechanism, we quantified key intermediates in the STX pathway. Spectrophotometric analysis demonstrated that SIL treatment significantly reduced the abundance of the downstream metabolites 4,4′-diaponeurosporene and 4,4′-diaponeurosporenic acid (Figure 7C). Concurrently, a marked accumulation of the pathway intermediate 4,4′-diapophytoene, the direct substrate of diapophytoene desaturase (CrtN), was observed. This metabolic profile, characterised by precursor accumulation and depletion of downstream products, strongly suggests that CrtN may represent a primary molecular target of SIL in the STX biosynthetic pathway. Definitive confirmation will require direct enzymatic assays using purified CrtN protein and genetic complementation studies in isogenic mutant strains.

## 3. Discussion

The medicinal plant *Silybum marianum*, renowned for its hepatoprotective constituent SIL, has recently attracted considerable interest owing to its broad pharmacological activities, including antimicrobial effects [19,20]. This study demonstrates that SIL exhibits both direct antibacterial and synergistic activity against *S. aureus*, while also displaying properties consistent with an antivirulence function, including inhibition of biofilm formation and, for the first time for a flavonolignan, reduction in STX biosynthesis.

The biocompatibility of SIL was confirmed by the absence of significant haemolytic activity in sheep erythrocytes and low cytotoxicity at sub-inhibitory concentrations in RAW 264.7 macrophages, with significant cytotoxic effects emerging only at concentrations ≥ 128 µg/mL. The synergistic combination of SIL and CIP did not induce significant cytotoxicity, indicating an adequate therapeutic window for their combined use. The direct antibacterial activity of SIL against *S. aureus* (MICs of 256–512 µg/mL) is weak to moderate, consistent with many plant-derived natural products, and modest compared with conventional antibiotics. As noted by Martelli and Giacomini (2018), naturally occurring compounds are typically classified as moderate inhibitors when their MICs are below 1000 µg/mL [25], a threshold that SIL meets. Rather than serving as a standalone systemic antibiotic, the value of SIL lies in its utility as a topical adjuvant, where high local concentrations can be achieved directly at the site of infection.

Synergistic interactions were observed in two of the four strains tested (FICI = 0.5 and 0.375 for ATCC 25923 and MRSA-4, respectively), whereas additive effects predominated in the remaining strains, indicating that SIL does not universally potentiate CIP activity. This strain-dependent pattern may reflect differences in the predominant resistance mechanisms across isolates and is consistent with the proposed inhibition of the NorA efflux pump by SIL [21]. MRSA-4 displayed high-level CIP resistance (MIC = 256 µg/mL), which was reduced fourfold upon co-administration with SIL, suggesting that this strain harbours an efflux-dominant resistance phenotype. In contrast, strains exhibiting only additive or indifferent effects may possess additional resistance mechanisms, such as target-site mutations in DNA gyrase or topoisomerase IV, which are not circumvented by efflux pump inhibition [26].

In addition to modulating antibiotic resistance, SIL also inhibits biofilm formation, a key virulence-associated phenotype. SIL significantly inhibited the formation of early-stage biofilms at sub-inhibitory concentrations and disrupted pre-formed mature biofilms in a strain-dependent manner. Notably, SIL monotherapy disrupted mature MRSA-4 biofilms at concentrations at or above its MIC (1×–4× MIC), whereas ciprofloxacin alone was ineffective. Given that mature biofilms typically require concentrations more than tenfold higher than those effective against planktonic cells for eradication [27], this finding supports the potential of SIL as a topical adjuvant, where high local concentrations can be achieved without systemic toxicity.

To investigate the molecular basis of these activities, molecular docking was performed on five clinically relevant targets: GyrB (fluoroquinolone target), DHFR (trimethoprim target), CrtM (STX biosynthesis), TMPK, and UPPs (cell wall biosynthesis). SIL exhibited binding energies comparable to those of reference inhibitors for GyrB (−8.703 vs. −8.465 kcal/mol for novobiocin) and CrtM (−10.539 vs. −10.742 kcal/mol for zaragozic acid A). All docking values represent computational estimates and require further experimental validation. These results generate testable hypotheses regarding the molecular targets of SIL. Substantiating a genuine multi-target mechanism would require, at a minimum, direct binding assays using purified recombinant proteins, functional inhibition assays for each individual target enzyme, and genetic studies employing single- and multiple-target deletion mutants to assess the contribution of each interaction to the observed biological effects of the compound. Until such evidence becomes available, the multi-target profile proposed here should be regarded as a computational hypothesis rather than a demonstrated pharmacological property of SIL. However, the most compelling mechanistic evidence derives from experimental metabolic profiling, which revealed, for the first time for a flavonolignan, that SIL inhibits the STX biosynthetic pathway. Spectrophotometric quantification of pathway intermediates demonstrated a significant reduction in downstream metabolites (4,4′-diaponeurosporene and 4,4′-diaponeurosporenic acid) alongside a concomitant accumulation of the precursor 4,4′-diapophytoene. This metabolic profile, characterised by precursor accumulation and depletion of downstream products, strongly suggests that CrtN may represent a primary target of SIL within the STX biosynthetic pathway. This metabolic signature mirrors the pattern reported by Chen et al. (2016) [28] and Gao et al. (2017) [29] to support CrtN inhibition, thereby providing indirect corroboration for this mechanistic hypothesis. Although molecular docking predicted favourable binding to CrtM (ΔG = −10.539 kcal/mol), CrtN is considered a particularly promising antivirulence target because, unlike CrtM, it lacks structural similarity to human squalene synthase, thereby mitigating potential safety concerns [30].

This study has several limitations. First, while the metabolic profiling data are consistent with CrtN inhibition, definitive confirmation requires direct enzymatic assays using purified CrtN and genetic complementation studies. Notably, CrtN was not included among the molecular docking targets in the present study, as the in silico analysis was designed prior to the identification of CrtN as the most strongly supported experimental target. Future computational analyses should specifically address the predicted binding affinity of SIL for this enzyme. Second, the docking results represent computational predictions that require experimental validation, and molecular dynamics simulations would further substantiate the stability of the predicted interactions under physiologically relevant conditions. The present study represents the initial stage of a structured mechanistic investigation (molecular docking → molecular dynamics simulations → binding free energy calculations → direct enzyme assays). These subsequent analyses will be reported separately due to their substantial computational and analytical demands. Accordingly, the docking results presented here are intended solely as hypothesis-generating, and all interpretations have been carefully calibrated to reflect this scope. Third, the predicted functional consequence of STX inhibition, namely increased susceptibility of *S. aureus* to reactive oxygen species generated by host neutrophils, was not directly assessed using oxidative stress killing assays (e.g., H_2_O_2_ survival experiments). Nonetheless, strong mechanistic evidence exists: unpigmented crtM mutants are significantly more susceptible to H_2_O_2_ and neutrophil-mediated killing [31], and multiple CrtN inhibitors have been shown to recapitulate this phenotype in murine infection models [28,29]. Fourth, the predominant resistance mechanisms of the individual strains included in the synergy assays were not characterised at either the genotypic or phenotypic level in the present study. Consequently, the mechanistic interpretation of the observed strain-dependent synergy pattern, including the proposed role of NorA-mediated efflux, remains hypothetical. Future studies should incorporate molecular characterisation of resistance determinants, such as *norA* expression levels and *gyrA/parC* sequencing, to prospectively identify strains that are more likely to benefit from SIL–CIP combination therapy.

Despite these limitations, the cumulative evidence supports the potential of SIL as a dual-function antivirulence agent. Future efforts should focus on in vivo validation, direct enzymatic assays, and targeted docking studies to confirm CrtN as a molecular target, as well as on the development of optimised topical formulations to translate these findings into therapeutic strategies for drug-resistant *Staphylococcus* infections.

## 4. Materials and Methods

### 4.1. Ligand and Target Preparation for Molecular Docking

The molecular structures of SIL and the reference compounds were modelled and visualised as three-dimensional (3D) representations using GaussView 5.0.8. The geometric and vibrational properties of these structures were computed in vacuo using density functional theory (DFT), employing the B3LYP hybrid functional in conjunction with the 6-31++G(d,p) basis set, as implemented in Gaussian 16 [32].

The three-dimensional (3D) structures of the selected targets—DNA gyrase B (GyrB), undecaprenyl pyrophosphate synthase (UPPs), dehydrosqualene synthase (CrtM), thymidylate kinase (TMPK), and dihydrofolate reductase (DHFR) from *S. aureus*—were retrieved from the Protein Data Bank (PDB) under the accession codes 4URO, 4H8E, 2ZCS, 4HLC, and 2W9H, respectively. These structures were determined by X-ray crystallography and exhibited acceptable structural quality. For the docking analysis, all non-essential molecules present in the crystal structures were removed, and the analysis was focused on the active site region.

### 4.2. Molecular Docking Studies

Molecular docking procedures were performed using AutoDock Vina 1.2.5 [33]. The structures of the selected targets and ligands were prepared using AutoDock Tools (ADT), version 1.5.7 [34]. In these simulations, the target structures were treated as rigid, whereas ligand flexibility was fully considered. Gasteiger partial charges were calculated following the addition of hydrogen atoms to both ligands and target structures. The dimensions of the cubic grid box were set to 30 × 30 × 30 Å along the X, Y, and Z axes, centred on the selected active-site residues: Pro87 for GyrB, Arg84 for UPPs, Tyr41 for CrtM, Arg48 for TMPK, and Phe92 for DHFR. The number of docking modes was set to 80, with an exhaustiveness level of 80 [35]. The conformations with the most favourable interaction energies for the ligand–receptor complexes were selected based on binding free energy, visual inspection, and analysis of the residues involved in ligand interactions. Molecular analysis and visualisation of the complexes were performed using the UCSF Chimera package [36] and PoseView [37].

Furthermore, redocking experiments were conducted on the PDB structures with their native ligands, following the same conditions and protocols applied to the studied compounds. The root mean square deviation (RMSD) was used to evaluate the predicted ligand positions from the docking simulations relative to their original positions in the crystal structures. This assessment was performed using Swiss-PdbViewer. The RMSD evaluation was critical for validating the accuracy and reliability of the docking methodology.

### 4.3. Bacterial Strains and Compounds

Four clinical isolates of *Staphylococcus aureus* were obtained from a research project approved by the Research Ethics Committee of the University Hospital of the Federal University of Maranhão (HU-UFMA) under protocol no. 7,846,824. Their antimicrobial susceptibility profiles are presented in Table S3 (Supplementary Materials). Reference strains (*S. aureus* ATCC 25923 and *S. aureus* ATCC 29213) were obtained from the culture collection of the Basic and Applied Microbiology Laboratory (BAML) at UFMA.

Silibinin (SIL) was obtained from Cayman Chemical (Ann Arbor, MI, USA) with a certified purity of ≥98% (HPLC). A stock solution of SIL was prepared by dissolving it in dimethyl sulphoxide (DMSO, Sigma-Aldrich, Saint Louis, MO, USA) and subsequently diluting it in Mueller–Hinton (MH) broth (Merck, Darmstadt, Germany), according to the manufacturer’s instructions. The solution was sterilised by filtration through a membrane with a pore size of 0.22 µm. Standard antimicrobials, ciprofloxacin (CIP) and oxacillin (OXA), were also obtained from Cayman Chemical. Individual stock solutions of each antimicrobial agent were prepared according to the manufacturer’s recommendations, aliquoted, and stored at −20 °C until use.

### 4.4. Determination of the Minimum Inhibitory Concentration of Silibinin

The minimum inhibitory concentration (MIC) was determined using the broth microdilution method, in accordance with the guidelines established by the Clinical and Laboratory Standards Institute [38]. Bacterial suspensions were prepared in sterile saline (0.85% NaCl) from fresh overnight cultures and adjusted to a turbidity equivalent to a 0.5 McFarland standard, yielding a suspension of approximately 1–2 × 10^8^ colony-forming units (CFU)/mL. The exact density of the initial suspension was confirmed by measuring the optical density at 625 nm (OD_625_). This standardised suspension was further diluted 1:100 in sterile Mueller–Hinton (MH) broth, resulting in a working inoculum of approximately 1–2 × 10^6^ CFU/mL. The assay was performed in sterile 96-well flat-bottom microplates (Kasvi, Pinhais, Brazil). To each well containing serial dilutions of the test compounds (SIL or antimicrobials), an equal volume of the working inoculum was added. This final 1:1 dilution resulted in a bacterial density of approximately 5 × 10^5^ to 1 × 10^6^ CFU/mL per well. SIL was tested over a concentration range of 32 to 1024 µg/mL. The following controls were included on each microplate: growth control (wells containing only the inoculum and MH broth), sterility control (MH broth only), and vehicle control (inoculum with the highest concentration of DMSO). The microplates were sealed and incubated at 37 °C for 18–20 h. After incubation, the MIC was visually determined as the lowest concentration of the compound that completely inhibited visible turbidity. A colourimetric resazurin assay was employed to provide an objective and sensitive endpoint. A 20 µL aliquot of a 0.01% (*w*/*v*) resazurin sodium salt solution was added to each well, and the plates were incubated at 37 °C for 2–4 h. A colour change from blue (oxidised) to pink or colourless (reduced) indicates metabolic activity and bacterial growth. The MIC was defined as the lowest concentration of the compound that prevented this colour change, confirming the absence of viable bacteria.

### 4.5. Determination of the Minimum Bactericidal Concentration of Silibinin

The minimum bactericidal concentration (MBC) was determined from wells showing no visible growth or resazurin conversion in the MIC assay. Aliquots (10 µL) from these wells were plated onto Mueller–Hinton agar plates, which were incubated at 37 °C for 24 h. The MBC was defined as the lowest concentration of the compound resulting in ≥99.9% killing of the initial inoculum, as evidenced by the absence of bacterial growth on subculture plates. The MBC/MIC ratio was calculated to classify the activity of SIL as bactericidal (MBC/MIC ≤ 4) or bacteriostatic (MBC/MIC > 4), according to established criteria [39].

### 4.6. Evaluation of In Vitro Interactions by Checkerboard Assay

The in vitro interaction between SIL and the antimicrobials (CIP and OXA) was investigated using the broth microdilution checkerboard method [40]. Assays were performed in sterile 96-well microplates. Antibiotics and SIL were serially diluted in twofold steps, ranging from 2 × MIC to 1/32 × MIC and from 2 × MIC to 1/128 × MIC, respectively. The final inoculum in each well was standardised to 1 × 10^7^ CFU/mL. The plates were incubated at 37 °C for 24 h.

The interaction was quantified by calculating the fractional inhibitory concentration index (FICI). The FIC of each agent was calculated as the MIC of the agent in combination divided by that of the agent alone, and the FICI was defined as the sum of the FICs of both agents.

FICI_index_ = FIC_SIL_ + FIC_Antibiotic_

where

FIC_SIL_ = (MIC of SIL in combination)/(MIC of SIL alone)

FIC_Antibiotic_ = (MIC of Antibiotic in combination)/(MIC of Antibiotic alone)

The FICI was interpreted as follows [41]:

- Synergy = FICI ≤ 0.5
- Additivity = 0.5 < FICI ≤ 1.0
- Indifference = 1.0 < FICI ≤ 4
- Antagonism = FICI > 4.0

Cell viability for FIC determination was confirmed using the resazurin assay, as described in Section 4.5. All assays were performed in triplicate and repeated in three independent experiments.

### 4.7. Effects of SIL, CIP, and Their Combinations on Biofilm Formation

#### 4.7.1. Interference with Early Stage Biofilm Formation

The effect of SIL on the formation of early-stage (nascent) biofilms was evaluated as previously described using sterile 96-well flat-bottom microplatess [42,43]. Biofilm adhesion was established by incubating 200 µL of a standardised inoculum (1 × 10^6^ CFU/mL) for 90 min at 37 °C. The wells were then washed three times with phosphate-buffered saline (PBS, Sigma-Aldrich, Saint Louis, MO, USA) to remove non-adherent cells, and 200 µL of treatment solutions diluted in MH broth were added at MIC and sub-inhibitory concentrations for *S. aureus* ATCC 25923 (256 to 64 µg/mL SIL, 0.5 to 0.125 µg/mL CIP, and FIC values ranging from 64 + 0.125 to 16 + 0.03 µg/mL) and MRSA-4 (512 to 128 µg/mL SIL, 256 to 64 µg/mL CIP, and FIC values ranging from 64 + 64 to 16 + 16 µg/mL SIL/CIP). Growth and sterility controls were included, and the plates were incubated at 37 °C for 24 h. Subsequently, the wells were washed three times with PBS and stained with MTT (3-(4,5-dimethylthiazol-2-yl)-2,5-diphenyltetrazolium bromide) [44]. Absorbance was measured using a UV–visible spectrophotometer at 570 nm.

#### 4.7.2. Pre-Formed Mature Biofilm

The effect of SIL on mature biofilms was analysed using a procedure similar to that described for early-stage biofilms up to the adhesion step [42,43]. After washing, 200 µL of MH broth was added, and the microplates were incubated for 24 h at 37 °C to allow biofilm maturation. The microplates were then washed three times with PBS and culture medium, followed by treatment with varying concentrations for *S. aureus* ATCC 25923 (256 to 1024 µg/mL SIL, 0.5 to 2 µg/mL CIP, and FIC values ranging from 64 + 0.125 to 256 + 0.5 µg/mL) and MRSA-4 (512 to 2048 µg/mL SIL, 256 to 1024 µg/mL CIP, and FIC values ranging from 64 + 64 to 256 + 256 µg/mL SIL/CIP). The plates were incubated for an additional 24 h at 37 °C and subsequently washed three times with PBS before assessing metabolic activity using an MTT assay. Absorbance was measured using a UV–visible spectrophotometer at 570 nm.

### 4.8. Investigation of the Inhibitory Effect of SIL on STX Biosynthesis

Quantification of STX production in MRSA-1 was performed using the methanol extraction method, as previously described [45,46], with minor modifications. Briefly, bacterial cells were re-inoculated at a 1:100 dilution in 15 mL of Tryptic Soy Broth (TSB, Difco, BD Diagnostics, Sparks, MD, USA) and incubated for 24 h at 37 °C, with or without SIL at sub-inhibitory concentrations (32, 64, and 128 µg/mL), in 50 mL tubes under shaking at 220 rpm. After incubation, the cells were collected by centrifugation (6000 rpm, 10 min, 4 °C) and washed with PBS. The resulting cell pellets were photographed to allow visual comparison of STX production. Methanol was subsequently used to extract STX from the cell pellets. In addition, the levels of STX biosynthesis intermediates were determined in response to SIL treatment. The absorbance of methanol-extracted carotenoids containing STX intermediates was measured using a microplate reader (BioTek Instruments, Winooski, VT, USA) at wavelengths of 286, 435, 455, and 465 nm, corresponding to 4,4′-diapophytoene, 4,4′-diaponeurosporene, 4,4′-diaponeurosporenic acid, and STX, respectively.

### 4.9. Haemolytic Assay

The assay was performed according to the methodology described by Silva et al. (2016) [47]. Sheep blood samples were provided by the Central Bioterium of UFMA (Brazil) and collected in tubes containing EDTA-K2 as an anticoagulant. Sheep blood (10 mL) was centrifuged at 1500 rpm for 5 min at 20 °C, and the plasma fraction was removed. The cell pellets were washed with an equal volume (10 mL) of PBS and gently mixed by inversion. The centrifugation and washing steps were repeated three times, after which the final volume was diluted 1:10 in PBS to obtain a suspension of approximately 5 × 10^8^ erythrocytes/mL. The assay was conducted in 96-well microplates. SIL (32–2048 µg/mL) and vehicle controls were tested using serial twofold dilutions in PBS. A 0.1% Triton X-100 solution was used as a positive control for haemolysis, while PBS served as the negative control. A volume of 100 µL of each test solution (SIL or diluted DMSO) was added to the wells, followed by 100 µL of the erythrocyte suspension, and incubated for 1 h at 37 °C. Subsequently, the contents were transferred to tubes and centrifuged at 1500 rpm for 5 min. An aliquot of 100 µL of the supernatant was then transferred to a new microplate and gently shaken at 1000 rpm for 1 min to eliminate air bubbles. Optical density was measured at 450 nm. The percentage of haemolysis was calculated using the following equation:

$$\%\;hemolysis=\;\frac{\left(SIL\;absorbance-PBS\;absorbance\;\right)\;\times 100}{\;\;\;(triton\;absorbance-PBS\;absorbance)}$$

### 4.10. Cytotoxicity Assay in Macrophages

The cytotoxic activity of the antibacterial agents (SIL, CIP, and SIL/CIP) was evaluated as previously described, with minor modifications [48]. Briefly, RAW 264.7 macrophages (2 × 10^6^ cells/mL) were cultured at 37 °C in a humidified atmosphere containing 5% CO_2_ and used for the in vitro cytotoxicity assay. Cells were treated with varying concentrations of SIL (32–2048 µg/mL), CIP (32–2048 µg/mL), and SIL/CIP (32 + 0.06 to 1024 + 2 µg/mL) in 96-well microplates. The negative control consisted of cells maintained in RPMI medium (Roswell Park Memorial Institute; Sigma-Aldrich^®^, São Paulo, Brazil) supplemented with 10% fetal bovine serum (FBS, Sigma-Aldrich, Saint Louis, MO, USA). The positive control consisted of cells treated with DMSO, and a medium-only control was also included. The plates were incubated for 48 h, after which they were centrifuged, and the supernatant was discarded and replaced with fresh medium containing MTT (5 mg/mL). The plates were then incubated for 3 h, centrifuged again, and the resulting formazan crystals were dissolved in 100 µL of pure DMSO. Absorbance was measured at 540 nm using a microplate reader (BioTek Instruments, Winooski, VT, USA).

### 4.11. Statistical Analyses

The data were subjected to the Shapiro–Wilk normality test (except for MIC, MBC, and in vitro interaction data). Significant differences between groups were determined by one-way analysis of variance (ANOVA), followed by Tukey’s multiple comparison test when normality assumptions were met. Statistical analyses were performed using GraphPad Prism software (version 8.0; GraphPad Software Inc., San Diego, CA, USA), and *p* < 0.05 was considered statistically significant. All experiments were conducted in triplicate across three independent experiments.

## 5. Conclusions

In conclusion, the findings of this study support the potential of SIL as a dual-function agent against *S. aureus*, exhibiting both direct antibacterial and antivirulence properties. Notably, this is the first report demonstrating that SIL inhibits staphyloxanthin biosynthesis at sub-inhibitory concentrations, with metabolic profiling suggesting interference with the CrtN-catalysed step, thereby disrupting a key virulence mechanism essential for immune evasion in *S. aureus.* SIL inhibited biofilm formation and disrupted mature biofilms of resistant MRSA isolates. Synergism with CIP significantly enhanced antibiotic efficacy against resistant strains. In silico analyses further suggested a potential multi-target mechanism, while experimental data more specifically indicated interference at the CrtN catalytic step within the STX biosynthetic pathway. Taken together, these findings support further investigation of SIL as a candidate topical antivirulence adjuvant. Future efforts should focus on in vivo validation and the development of optimised formulations to translate these findings into practical therapeutic strategies for drug-resistant staphylococcal infections.

## Figures and Tables

**Table 1:** Minimum inhibitory and bactericidal concentration assays for reference and clinical bacterial strains.

Strains *	MIC SIL **	MBC SIL **	MBC/MIC ***	Activity ***
*S. aureus* ATCC 29213	512	>1024	-	-
*S. aureus* ATCC 25923	256	512	2	Bactericidal
MRSA-1	512	>1024	-	-
MRSA-2	512	1024	2	Bactericidal
MRSA-3	256	>1024	-	Bacteriostatic
MRSA-4	512	>1024	-	-

* ATCC, American Type Culture Collection strains; MRSA-1, MRSA-2, MRSA-3, and MRSA-4 represent clinical isolates of *S. aureus.* ** Values expressed in µg/mL, *** MBC/MIC ratios: Activity was classified as bactericidal when MBC/MIC ≤ 4 and bacteriostatic when MBC/MIC > 4. “-” (not detected) indicates cases where the MBC exceeded the highest tested concentration (>1024 µg/mL), meaning that the activity could not be classified based on the MBC/MIC ratios.

**Table 2:** Interactions between SIL and antibiotics against reference and clinical bacterial strains.

Strains *	Drug	MIC (µg/mL)		FIC	FICI	Effect **
		Isolated	Combined			
*S. aureus* ATCC 29213	SIL	512	16	0.031	0.531	AD
	CIP	0.5	0.25	0.5		
	SIL	512	256	0.5	1	AD
	OXA	0.5	0.25	0.5		
*S. aureus* ATCC 25923	SIL	256	64	0.25	0.5	SYN
	CIP	0.5	0.125	0.25		
	SIL	256	512	2	2.5	IND
	OXA	0.25	0.25	0.5		
MRSA-2	SIL	512	16	0.031	0.531	AD
	CIP	16	8	0.5		
	SIL	512	256	0.5	1	AD
	OXA	64	32	0.5		
MRSA-4	SIL	512	64	0.125	0.375	SYN
	CIP	256	64	0.25		
	SIL	512	256	0.5	1	AD
	OXA	256	128	0.5		

* ATCC (American Type Culture Collection) strains; MRSA-2, and MRSA-4 were clinical isolates of *S. aureus*; ** Synergism (SYN): ΣFICI ≤ 0.5; additive (AD) 0.5 < Σ FICI ≤ 1; indifference (IND) 1 < Σ FICI ≤ 4.0; and Antagonism (AN): Σ FICI > 4.0.

**Table 3:** Molecular docking binding affinities of SIL and reference compounds against *S. aureus* targets.

Ligand	Target/ΔGbind (kcal/mol)				
	GyrB	UPPs	CrtM	TMPK	DHFR
	(#4URO)	(#4H8E)	(#2ZCS)	(#4HLC)	(#2W9H)
Silibinin	−8.703	−8.701	−10.539	−8.542	−11.840
novobiocin *	−8.465	-	-	-	-
ciprofloxacin #	−7.621	-	-	-	-
farnesyl diphosphate *	-	−8.283	-	-	-
viridicatumtoxin #	-	−8.231	-	-	-
B70 *	-	-	−10.581	-	-
zaragozic acid A #	-	-	−10.742	-	-
T05 *#	-	-	-	−9.973	-
trimethoprim *#	-	-	-	-	−14.840

* redocking from native structure crystal ligand; #: positive control; “-”: not rated.

## Data Availability

The original contributions presented in this study are included in the article/Supplementary Material. Further inquiries can be directed to the corresponding authors.

## Supplementary Materials

The following supporting information can be downloaded at: https://www.mdpi.com/article/10.3390/ph19040643/s1, Table S1: CIP MIC and MBC against *Staphylococcus aureus* strains; Table S2: OXA MIC and MBC against *Staphylococcus aureus* strains; Table S3: Antibiotic susceptibility profiles of clinical isolates of *Staphylococcus aureus.*

## Abbreviations

The following abbreviations are used in this manuscript:

ADT	Autodock Tools
ATCC	American Type Culture Collection
BAML	Basic and Applied Microbiology Laboratory
CIP	Ciprofloxacin
CrtM	Dehydrosqualene Synthase
CrtN	Diapophytoene Desaturase
DFT	Density Functional Theory
DHFR	Dihydrofolate Reductase
DMSO	Dimethyl Sulfoxide
FBS	Fetal Bovine Serum
FIC	Fractional Inhibitory Concentration
FICI	Fractional Inhibitory Concentration Index
GyrB	DNA Gyrase B
HPLC	High-Performance Liquid Chromatography
MBC	Minimum Bactericidal Concentration
MIC	Minimum Inhibitory Concentration
MRSA	Methicillin-Resistant *Staphylococcus aureus*
MTT	3-4,5-dimethyl-thiazol-2-yl-2,5-diphenyltetrazolium bromide
OXA	Oxacillin
PBS	Phosphate-Buffered Saline
PDB	Protein Data Bank
RMSD	Root Mean Square Deviation
SIL	Silibinin
STX	Staphyloxanthin
TSB	Tryptic Soy Broth
TMPK	Thymidylate Kinase
UFMA	Federal University of Maranhão
UPPs	Undecaprenyl Pyrophosphate Synthase
WHO	World Health Organization
