# Supplementary material for: Silibinin Potentiates Antimicrobial Action and Reduces Staphyloxanthin in Staphylococcus aureus
Source: Pharmaceuticals (Basel). 2026 Apr 18;19(4):643. doi: 10.3390/ph19040643 (PMC13119222; doi:10.3390/ph19040643)
Supplement: Supplementary file 1 [file pharmaceuticals-19-00643-s001.zip › pharmaceuticals-4213591-supplementary.pdf]

Silibinin Potentiates Antimicrobial Action and Reduces Staphyloxanthin in *Staphylococcus aureus*

José Lima Pereira-Filho[1], Amanda Graziela Gonçalves Mendes[1], Carmem Duarte Lima Campos[1], Viviane da Silva Sousa Almeida[1], Aleania Polassa Almeida Pereira[1], Israel Viegas Moreira[1], Cinara Regina Aragão Vieira Monteiro[2], Louriane Nunes Gomes[3], Cristianne Roberta Rhoden[4], Antonio José Cantanhede-Filho[5], Lucilene Amorim Silva[3], Alberto Jorge Oliveira Lopes[5*], Rafael Cardoso Carvalho[1], and Valério Monteiro-Neto[1*]

[1] Graduate Program in Health Sciences, Federal University of Maranhão – UFMA, São Luís 65080-805, MA, Brazil

[2] School of Physical Therapy, Florence University Center, São Luís 65.020-490, MA, Brazil

[3] Laboratory of Pathology and Immunoparasitology, Center of Biological and Health Sciences, Federal University of Maranhão, São Luís, MA, 65080-805, Brazil

[4] Cedro Laboratory, São Luís, MA, 65020-570, Brazil

[5] Graduate Program in Chemistry, Federal Institute of Scientific and Tecnological Education of Maranhão, São Luís, MA, 65030-005, Brazil

Supporting Information

Content | **Page**

**Table S1**	CIP MIC and MBC against *Staphylococcus aureus* strains	3
**Table S2**	OXA MIC and MBC against *Staphylococcus aureus* strains	4
**Table S3**	Antibiotic susceptibility profiles of clinical isolates of *Staphylococcus aureus*	5

Table S1. CIP MIC and MBC against *Staphylococcus aureus* strains.

Strains*	MIC CIP **	MBC CIP **	MBC/MIC ***	Activity ***
S. aureus ATCC 29213	0.5	0.5	1	Bactericidal
S. aureus ATCC 25923	0.5	0.5	1	Bactericidal
MRSA-1	16	32	2	Bactericidal
MRSA-2	16	256	16	Bacteriostatic
MRSA-3	2	8	4	Bactericidal
MRSA-4	256	>1.024	-	Bacteriostatic

*ATTC, American Type Culture Collection strains, MRSA-1, MRSA-2, MRSA-3, and MRSA-4, represent clinical isolates of *S. aureus*. **Values expressed in µg/mL, ***MBC/MIC ratios: Activity was classified as bactericidal when MBC/MIC ≤ 4 and bacteriostatic when MBC/MIC > 4. "-" (not detected) indicates cases where the MBC exceeded the highest tested concentration (>1024 µg/mL), meaning that the activity could not be classified based on the MBC/MIC ratios.

Table S2. OXA MIC and MBC against *Staphylococcus aureus* strains.

Strains*	MIC OXA**	MBC OXA **	MBC/MIC ***	Activity***
S. aureus ATCC 29213	0.5	0.5	1	Bactericidal
S. aureus ATCC 25923	0.25	0.25	1	Bactericidal
MRSA-1	128	128	1	Bactericidal
MRSA-2	64	128	2	Bactericidal
MRSA-3	64	128	2	Bactericidal
MRSA-4	256	256	1	Bactericidal

*ATTC, American Type Culture Collection strains, MRSA-1, MRSA-2, MRSA-3, and MRSA-4, represent clinical isolates of *S. aureus*. **Values expressed in μg/mL, ***MBC/MIC ratios: Activity was classified as bactericidal when MBC/MIC ≤ 4 and bacteriostatic when MBC/MIC > 4.

Table S3. Antibiotic susceptibility profiles of clinical isolates of *Staphylococcus aureus*.

Antibiotics	Clinical isolates*			
	**MRSA-1**	**MRSA-2**	**MRSA-3**	**MRSA-4**
Amikacin	S	-	-	S
Ampicillin	R	-	-	R
Amoxicillin-Clavulanate	R	-	-	R
Cefepime	-	-	-	-
Ceftazidime	-	-	-	-
Ceftriaxone	-	-	-	-
Cephalothin	S	-	-	S
Cefazolin	-	-	-	-
Cefotaxime	-	-	-	-
Ciprofloxacin	R	R	I	R
Clindamycin	R	S	S	R
Imipenem	-	-	-	-
Ertapenem	-	-	-	-
Erythromycin	R	R	-	R
Gentamicin	S	S	S	S
Levofloxacin	-	R	I	-
Linezolid	S	S	S	-
Meropenem	-	-	-	-
Minocycline	R	-	-	-
Norfloxacin	-	-	-	R
Oxacillin	R	R	R	R
Penicillin G	R	-	-	R
Piperacillin/ Tazobactam	-	-	-	-
Polymyxin B	-	-	-	-
Rifampicin	S	S	-	-
Sulfamethoxazole/ Trimethoprim	S	S	S	-
Teicoplanin	S	S	-	-
Tetracycline	S	-	-	S
Vancomycin	S	S	S	S

MRSA: Methicillin-resistant *Staphylococcus aureus*; R: resistant; S: sensitive; I: intermediate.
